# Modelling Lipid Competition Dynamics in Heterogeneous Protocell Populations

**DOI:** 10.1038/srep05675

**Published:** 2014-07-14

**Authors:** Ben Shirt-Ediss, Kepa Ruiz-Mirazo, Fabio Mavelli, Ricard V. Solé

**Affiliations:** 1 ICREA-Complex Systems Lab, Institut de Biologia Evolutiva, CSIC-UPF, Barcelona, Spain; 2 Logic and Philosophy of Science Department, University of The Basque Country, Spain; 3 Biophysics Unit (CSIC-UPV/EHU), University of The Basque Country, Spain; 4 Chemistry Department, University of Bari, Italy; 5 Santa Fe Institute, 1399 Hyde Park Road, Santa Fe NM 87501, USA

## Abstract

Recent experimental work in the field of synthetic protocell biology has shown that prebiotic vesicles are able to ‘steal’ lipids from each other. This phenomenon is driven purely by asymmetries in the physical state or composition of the vesicle membranes, and, when lipid resource is limited, translates directly into competition amongst the vesicles. Such a scenario is interesting from an origins of life perspective because a rudimentary form of cell-level selection emerges. To sharpen intuition about possible mechanisms underlying this behaviour, experimental work must be complemented with theoretical modelling. The aim of this paper is to provide a coarse-grain mathematical model of protocell lipid competition. Our model is capable of reproducing, often quantitatively, results from core experimental papers that reported distinct types vesicle competition. Additionally, we make some predictions untested in the lab, and develop a general numerical method for quickly solving the equilibrium point of a model vesicle population.

A fundamental problem in biology concerns the origins of an innovation that allowed the development of organisms in our biosphere, beyond complex chemical reaction networks: the emergence of cells^1^,^2^. Cells define a clear scale of organization and, given their spatially confined structure, they constitute efficient units where molecules can easily interact, coordinate their dynamical patterns and establish a new level of selection. Although it is often assumed that there was a transition from some type of ‘less-organised’ prebiotic chemistry (probably including catalytic cycles) to a cell-based living chemistry, little is yet known about the potential pathways that could be followed to cross it. Once in place, protocell assemblies would require available resources for their maintenance and, thus, would naturally get inserted in diverse competitive dynamics in which the main selective unit would be the whole protocellular system. In this context, aggregate-level evolution is the right scale of analysis to be considered.

Different types of protocellular systems of diverse complexity have been studied from a theoretical standpoint^3^,^4^,^5^,^6^,^7^,^8^,^9^,^10^,^11^. In particular, by considering the coupling of a template carrying information with vesicle replication and metabolism, it has been shown that Darwinian selection is the expected outcome of competition in a protocellular world^12^. In a more simple scenario for autopoietic (i.e. self-producing) vesicles in a homeostatic regime, previous numerical simulations suggest that random fluctuations can also act as ‘selection rules’ for the more robust individuals^13^. Early pre-Darwinian stages in the development of biological organisms in which supramolecular systems could still be disconnected from information (i.e. closer to elementary forms of metabolism and strongly constrained by the molecular diversity of the available chemical repertoire) ought to be further explored. What type of competition and cooperation processes were at work in the chemical world leading to the emergence of early protocells? Processes able to favour asymmetries in the chemical composition of vesicles should be expected to play a relevant role in this context, defining the conditions under which protocellular assemblies could thrive.

Recent laboratory experiments have actually demonstrated how differences in the composition or physical state of the vesicle membrane can drive competition for simple amphiphilic molecules (typically fatty acids), present in solution as free monomers at low concentration. First, Cheng and Luisi^14^ observed competition between pure oleic acid and POPC vesicles, where each of these vesicle populations had different initial size distributions. In all the studied cases, the final size distribution was found near to the initial one of the POPC vesicles, suggesting that oleic acid molecules were rapidly absorbed by POPC aggregates. Then, Chen et al.^15^ reported competitive dynamics in a population of fatty acid vesicles, whereby vesicles that were osmotically swollen by an encapsulated cargo of RNA (or sucrose) stole lipids from their empty, osmotically relaxed counterparts by virtue of absorbing monomers more quickly from the solution. They studied both oleic acid and POPC vesicles, but only in the former case was competition observed. This distinctive behaviour of fatty acid vesicles has been theoretically rationalised^16^ by assuming that double chain phospholipids are taken up from solution by the vesicle membranes five orders of magnitude more slowly than single chain fatty acid molecules.

More recent experimental work has turned attention to other possible selective advantages of protocells, such as phospholipid-17 and peptide-18 driven competition amongst vesicles. Instead of membrane tension, the main factor for competition here is the different composition of the membrane; single-chain fatty acids are mixed with double chain amphiphiles or with a different type of surfactant molecule, like sufficiently hydrophobic peptides. In the case of phospholipid-driven competition, oleic acid vesicles endowed with a membrane fraction of phospholipid are observed to take fatty acid molecules from phospholipid-deficient neighbours, who shrink, whilst the former grow and keep their potential for reproduction.

In this paper we develop a mathematical model of a competing population of vesicles, with the motivation to explore and test possible mechanisms underlying lipid competition phenomena. The model is based at the coarse-grain level of lipid kinetics, following the approach of Mavelli and Ruiz-Mirazo^16^. Using physically realistic parameters such as lipid molecule sizes, vesicle aggregation numbers and critical vesicle concentrations (CVC) as detailed in Table 1, we are able to qualitatively and often quantitatively reproduce results from two key experimental papers describing phospholipid-driven^17^ and osmotically-driven^15^ competition.

In the model, a vesicle in a population absorbs and releases amphiphiles to and from its membrane at rates that depend on the current physical properties of that particular vesicle (such as membrane composition or extent of osmotic tension). To take account of phospholipid-driven competition, we build into the lipid kinetics two basic physical mechanisms, which have been postulated in the literature to underlie asymmetric growth dynamics in this context: the *indirect effect* and the *direct effect*, as will be named in this work. The first one refers to the decrease of amphiphile release processes simply due to the fact that other surfactant molecules are present in the membrane, and the second to the immediate influence that these surfactant molecules could have on the amphiphiles (see Fig. 1).

More generally, this work forms part of our endeavour to try to develop a formalism that grasps the lipid kinetics involved in vesicle self-assembly under controlled conditions (pH, temperature, etc.). In contrast to the kinetics of chemical reaction networks which have been extensively modelled by the Mass Action Kinetics (MAK) and Stochastic frameworks^19^,^20^, membrane lipid kinetics have been largely under-explored in the literature, due to the inherent complexity of supramolecular structures. Nevertheless, models coupling together membrane and metabolism kinetics will be a crucial cornerstone in order to build a systems understanding of the dynamic properties and organization of protocells, ultimately biological cells as well.

The paper is organised as follows. The remainder of the introduction serves to both introduce our kinetic model in detail, and to perform a mean-field analysis of it. This analysis gives insight into why we should expect phospholipid-driven competition to result in a simplified version of our model. Then, the Results section summarises how well numeric simulations of the full kinetic model are able to reproduce experimental results and observations, including also some predictions for still untested protocell competition scenarios. In the Discussion section, we comment on some assumptions and other aspects of our approach and conclude the study. The Methods section at the end of the paper describes a fast numerical method for solving the final equilibrium state of the full model. This method was essential in producing the results figures in the paper. The Supplementary Material (online) explains aspects in more detail, including justification for some modelling choices and the vesicle mixing procedure assumed in order to compare our model with experimental observations.

## Theoretical model of vesicle competition

**Figure 2:**
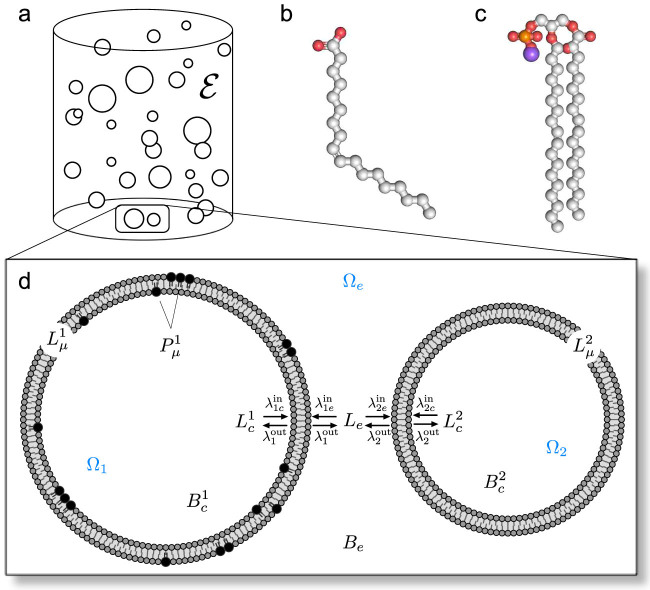
Kinetic model of vesicle competition. (a) Our model approach considers as a starting point a population of vesicles (of generally heterogeneous sizes and membrane compositions) in a well-mixed environment. (b) Each vesicle has a membrane composed of simple single chain lipids *L*, e.g. oleic acid (OA), and (c) sometimes more complex double chain phospholipids *P*, e.g. dioleoyl-phosphatidic acid (DOPA). (d) outlines the kinetic interactions between vesicles. Here two vesicles are displayed (bilayer cross sections not to scale). Vesicle 1, on the left hand side, has a mixed membrane with approximately 10 mol% phospholipids *P* (black) and the remainder single chain lipids *L* (grey). Vesicle 2 consists purely of simple lipids *L*. In the ensuing competition, phospholipid-laden vesicle 1 will grow at the expense of vesicle 2, which will shrink.

The competition model (Fig. 2) involves a set of *n* vesicles 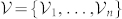, each one characterized by a quintuple 
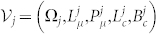
 and all embedded in a finite volume environment 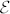 defined by a triple (Ω*_e_*, *L_e_*, *B_e_*).

Each competing vesicle 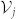 consists of a unilamellar (single bilayer) membrane of up to two different lipid types: single-chain fatty acid lipids 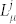 (e.g. oleic acid, OA), and possibly a fixed number of double-chain phospholipids 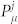 (e.g. di-oleoyl-phosphatidic acid, DOPA). Membrane thickness is considered negligible, and surface area of a vesicle, referred to as 
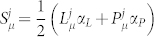
, is the water-exposed area of either of the bilayer leaflets. The *L* type lipids in the bilayer continuously exchange with the vesicle internal water pool and 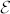, whereas the phospholipids *P* are considered approximately stationary in the bilayer due to their comparatively slow exchange rate, in agreement with previous reported work using POPC vesicles^16^. The internal water pool of each vesicle is considered a well-mixed chemical domain of volume Ω*_j_* and hosts 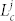 lipid monomers and also 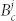 buffer species. Buffer species cannot permeate the bilayer but provide osmotic stability and they are also present in 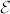 with constant number *B_e_*.

Vesicles compete with each other by consequence of uptaking/releasing fatty acid monomers *L* from/to 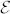, which is a common limited resource. The initial system of vesicles is taken to be the result of mixing different vesicle populations, and is a closed system in a non-equilibrium state. The system equilibrates to a final state following the dynamics described below, with some vesicles growing bigger in surface at the expense of others, which shrink. We ignore spatial correlations and the possibility of direct vesicle-vesicle interactions, and assume a well-mixed set of vesicles.

More precisely, each vesicle 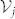 is considered to release lipids to both aqueous phases (at each side of the bilayer) at the equal rate of 
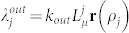
, and absorb lipids from each phase at rate 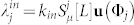, where [*L*] is the molar concentration of lipid monomer in the respective phase. Functions **r** and **u** are defined later.

The uptake and release kinetics are symmetric on each side of the bilayer, which means that the lipid monomer concentration inside and outside each vesicle will be equal 
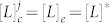
 at equilibrium. Flip-flop of the fatty acid *L* between membrane leaflets is considered very fast with respect to its uptake and release rates, and thus a bilayer is modelled as a single oily phase; this simplification is supported by experimental work from Hamilton's lab^21^,^22^. Conceivably, leaflet asymmetries could be created by the fact that the flip-flop of deprotonated and protonated fatty acid molecules is not the same^23^. However, such effects are considered of secondary importance and are disregarded in the present work.

Explaining the choice of *L* release kinetics, each fatty acid in a pure *L* membrane is considered to have a uniform probability per unit time *k_out_* of disassociating from the membrane^16^, while function **r** has been introduced in this work to take into account the *direct effect*. This function (0 ≤ **r**(*ρ*) ≤ 1) modifies the fatty acid release probability, based on the current molecular fraction of phospholipid *P* in a membrane 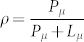. It is monotonically decreasing with increasing *ρ*, meaning that increasing phospholipid fraction generally decreases bilayer fluidity, slowing down the rate of *L* release from the membrane^17^. In a first approximation, **r** was assumed linear: 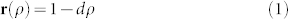
where parameter 0 ≤ *d* ≤ 1 tunes how the lipid release rate is affected by phospholipid content (1 being maximally affected and 0 being not at all).

Conversely, lipid uptake kinetics reflect that the probability of uptaking a lipid *L* to the membrane is proportional to the density of lipid monomer in the immediate vicinity of the respective bilayer surface (i.e. the concentration of lipid in the surrounding medium), the area of surface available for absorption *S_μ_* and function **u**, based on the dimensionless *reduced surface* 
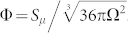
 The reduced surface encodes the surface area to volume ratio of a vesicle, when the latter internal volume is considered as a sphere: Φ = 1 denotes a vesicle perfectly spherical in shape, whereas Φ < 1 or Φ > 1 indicates a vesicle in osmotic tension or deflated respectively. Taking this into consideration, we define **u** as the following conditional function 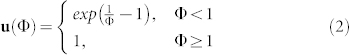
to denote that lipid uptake is only increased when the the bilayer is stressed^24^. Flaccid vesicles do not have extra enhancement of lipid uptake rate. Rationale for this function originated in the theoretical modelling of osmotically-driven competition dynamics between fatty acid vesicles^15^,^16^, and additional justification is provided in the Supplementary Material.

The *indirect effect* is manifest as a systems property of the model, rather than in any particular function. When a vesicle membrane contains phospholipids (or other surfactant species like hydrophobic peptides), the *P_μ_* molecules add a contribution to the surface, increasing the *L* uptake rate, whereas the *L* release rate remains unaffected by their presence.

Uptake and release kinetic constants *k_in_* and *k_out_* are set by two criteria. The first criterion is that pure fatty acid model vesicles (made solely of *L*), either spherical or deflated, must be in equilibrium when the fatty acid monomer concentration inside and outside the vesicle is the CVC for that amphiphilic compound (e.g. oleic acid). The second criterion is that the model dynamics must reproduce, with lowest RMS error, the experimental time courses reported by Chen et al.^15^ for surface changes in osmotic competition. The second criterion narrows the possible {*k_in_*, *k_out_*} pairs (see Supplementary Material). For mixed membrane vesicles containing both *L* and *P* lipids, we assume that the lipid kinetics equations define what lipid monomer concentration inside and outside the vesicle [*L*]*_eq_* is necessary to keep the mixed membrane vesicle in equilibrium (however, in reality, the CVC of mixed lipid solutions is not a trivial matter^25^).

For the purpose of lipid competition, 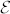 has a fixed volume of Ω*_e_* litres. Each vesicle 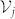 has, in principle, a variable internal water volume of 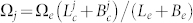 litres. This volume value is based on the assumption that water permeates the membrane extremely rapidly, and ensures that the interior of each vesicle is isotonic with respect to 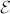 at all times. However, since in real fatty acid vesicle solutions the concentration of extra compounds like counter ions, pH buffer, etc., is much higher than the concentration of free fatty acid monomers, we can reasonably assume that the aqueous volume of each vesicle is approximately constant at 
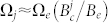
. Thus, vesicle volume is largely determined by the number of buffer molecules a vesicle has trapped inside its internal water pool, with *L* flux to and from the water pool having marginal osmotic effects.

To summarise, the state of the vesicle system is captured by enumerating the number of lipids in each of the aqueous pools inside the vesicles, and in each of the vesicle membranes. The ODE system that describes the time behaviour of the entire vesicle population consists of 2*n* equations, two for each vesicle: 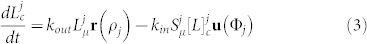

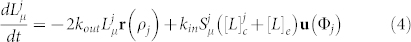


At the same time, the total number of lipids in the system *L_t_* is a conserved quantity set by the initial condition of mixing (see Supplementary Material), always equal to the number of lipid monomers in the environment *L_e_*, plus the number of lipids composing the vesicles: 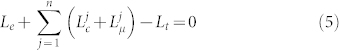
Therefore, *L_e_* can be deduced from equation (5) once all *L_c_* and *L_μ_* have been calculated at time *t*. Values of model parameters are given in Table 1.

## Mean field approximation

**Figure 3:**
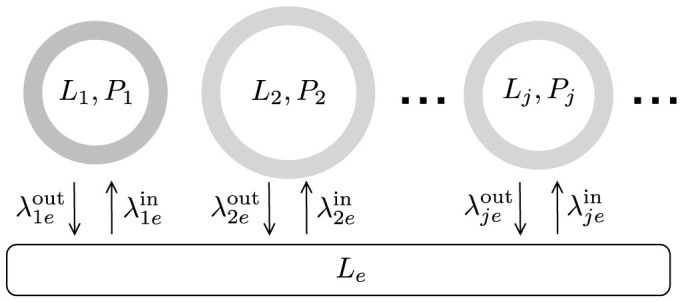
Meanfield model of vesicle population dynamics. Some analytical treatment of the model is possible if vesicles lose their internal structure, and are just considered to exchange fatty acid with the external solution following simplified kinetic rate equations.

In the first instance, before performing any numeric simulations, why should we expect phospholipid fraction and surface growth to be correlated in the vesicle competition model? To answer this question, we can make a mean field approximation. This approach considers a reduced scenario where many details associated to the full model are ignored in order to keep only the logic of the problem (Fig. 3).

The first simplification will be to ignore the internal structure of the vesicles, describing them instead as coarse-grained ‘aggregates’, denoted by pairs 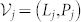, which contain just lipids and phospholipids. This step can be considered justified on the grounds that, at equilibrium, the amount of lipid monomer residing in the vesicle water pools (which typically have tiny volumes, around 1 quintillionth of a litre) is marginal as compared to the lipid composing the vesicle membranes. Since the internal structure or topology of the vesicles is disregarded, it actually amounts to treating them as elongated micelles or flat bilayers.

The second simplification involves reducing the lipid uptake and release equations to their most basic form, independent of membrane tension (**u**(Φ) = 1) and independent of membrane phospholipid fraction (**r**(*ρ*) = 1) respectively. Thus, the ODE system reduces to *n* simplified equations, where for each aggregate: 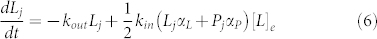
Under these conditions, at equilibrium, the molar lipid concentration in the environment [*L*]*_e_* = [*L*]*_eq_* is related to the number of lipids and phospholipids in an aggregate by the following function: 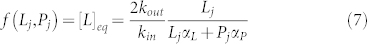
For a fixed number of phospholipids *P_j_* > 0, the mapping *f*:*L_j_* → [*L*]*_eq_* can be verified to be one-to-one, meaning that each aggregate is in equilibrium at only one specific outside lipid concentration, dependent on the number of lipids *L_j_* it contains. Thus, no multiple equilibria of the population are allowed from this type of aggregate dynamics.

Now consider two arbitrarily chosen aggregates *i* and *j* in the population of *n* aggregates, which are competing for lipid. Their ODEs, when written as: 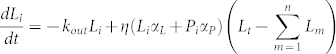

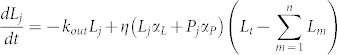
where *η* = *k_in_*/2*N_A_*Ω*_e_*, are reminiscent of the Lotka-Volterra competition equations associated to species sharing and competing for a common set of resources^26^. If we look for the equilibrium solutions of the previous system, using *dL_i_*/*dt* = *dL_j_*/*dt* = 0, we obtain 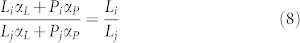
which leads to the following proportionality relation at equilibrium: 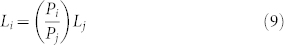
meaning that the final equilibrium vesicle sizes will be correlated with their respective numbers of phospholipids. Unless *P_i_* = *P_j_* one of the vesicles will be larger and the second smaller. For each pair (*P_i_*, *P_j_*) with *P_i_* ≠ *P_j_* a single solution is found.

When functions **u** and/or **r** are not constant, unless they have a trivial form, it is generally not possible to show analytically what shape the correlation between phospholipid fraction and surface growth will take. However, in the Methods section at the end of the paper, we develop a fast numerical way to find the equilibrium configuration of the fully-fledged vesicle population model, with vesicles recovering their internal structure. As compared to numerically integrating the ODE set, the method provides the extra advantages of (i) being faster and thus scaling better for large vesicle populations and (ii) being able to calculate competition ‘tipping points’ (i.e. critical points that mark the transition between growing and shrinking) directly.

In the following Results section, our fast procedure was used to perform accurate vesicle stoichiometry calculations, whilst simulations of the model dynamics were carried out using small populations of vesicles and deterministically integrating the ODE set.

## Results

### Two competing populations: comparison with experimental results

**Figure 4:**
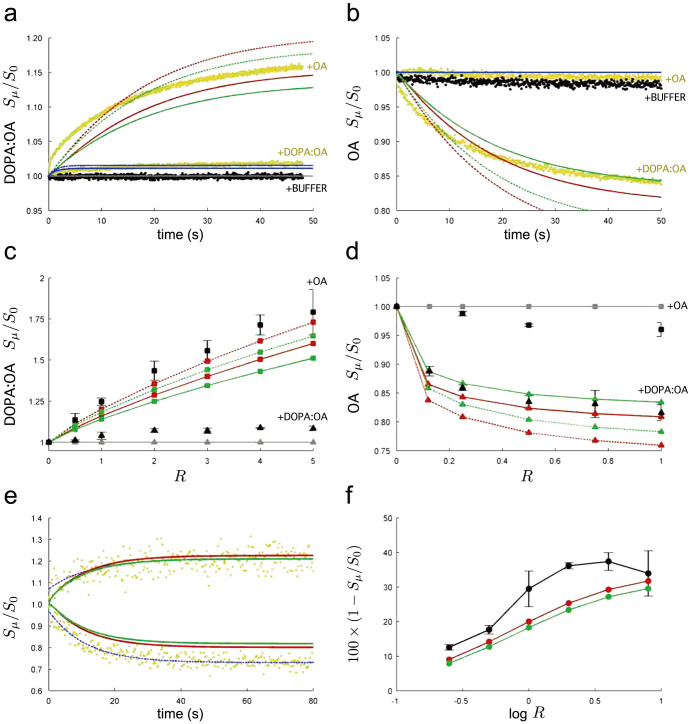
Comparison between kinetic model predictions and experimental results. In all plots, model vesicles extruded with 120 nm diameter surface area, either being spherical (Φ = 1, green lines) or deflated by 5% (Φ = 1.0348, red lines). Solid lines denote indirect effect only (*d* = 0) and dotted lines denote maximal direct effect (*d* = 1) present in DOPA:OA vesicles. Top plots show *dynamics* of phospholipid-driven competition. Experimental data points from Budin & Szostak^17^ (Figs. 1A, 1B therein) reproduced in background as coloured dots. (a) Surface change of model DOPA:OA (*ρ*_0_ = 0.1) vesicles over time when mixed 1:1 with pure OA vesicles, with similar DOPA:OA (*ρ*_0_ = 0.1) vesicles, and with buffer. Model outcomes when mixing with similar DOPA:OA vesicles shown as horizontal grey line, and when mixing with buffer, as blue lines. (b) Surface change of pure OA vesicles over time when mixed 1:1 with the same three options as in (a). Model outcome when mixing with OA vesicles or with buffer shown as horizontal blue line. Middle plots show *vesicle stoichiometry effects* in phospholipid-driven competition. Supplementary Material defines our interpretation of vesicle mix ratio *R* in detail. (c) Continued average surface growth in fixed population of model DOPA:OA vesicles as more OA vesicles added at increasing mix ratio *R* and (d) plateau of average surface shrinkage in fixed population of OA vesicles as more DOPA:OA vesicles added at increasing mix ratio *R*. Black markers with error bars reproduce experimental data points from Budin & Szostak^17^ (Figs. 1C, 1D therein). Bottom plots show osmotically-driven competition results. (e) Growth dynamics of model swelled OA vesicles and shrinkage of isotonic OA vesicles compared against best-fit exponential decay curves (dotted blue lines) to experimental data points from Chen et al.^15^ (Figs. 1D, 1B therein). (f) Stoichiometry effects in osmotically-driven competition. Shrinkage of OA vesicle surface reaches a plateau as more swelled vesicles are added at increasing mix ratio *R* (note log scale). Black line and markers reproduce experimental results from Chen et al.^15^ (Fig. 2A therein). Minimum Φ reached by model OA vesicles in (a)–(d) is 0.7692, in (e) is 0.7046.

Figure 4 compares predictions made by our kinetic model against experimentally reported surface growth of vesicles (assessed by a Förster resonance energy transfer assay, FRET) in phospholipid-driven^17^ and osmotically-driven^15^ competition. Two scenarios are forecast by our model: one where vesicles are spherical when extruded, and one where they are deflated by 5% when extruded, as generally observed experimentally^24^,^27^. The Supplementary Material details the vesicle population mixing procedure used to initialise our theoretical model, adjusting it to realistic experimental conditions.

Top figures 4a and 4b show the *dynamics* of surface area change in phospholipid-driven competition. Figure 4a details, in real time, the relative surface area of a tracked (surface area followed by fluorescence probe) population of DOPA:OA (*ρ*_0_ = 0.1) vesicles, when this population is mixed 1:1 with either pure OA vesicles, similar DOPA:OA (*ρ*_0_ = 0.1) vesicles or simply buffer. In Fig. 4b, the tracked population is instead pure OA vesicles, which are mixed 1:1 with the same three options outlined above.

Whether starting with initially spherical vesicles, or vesicles deflated by 5%, execution of our lipid kinetics model correctly predicts that when mixed 1:1, DOPA:OA vesicles steal lipid and grow (rising lines, Fig. 4a) at the expense of the pure OA vesicles, which shrink (falling lines, Fig. 4b). In this case, there is also fairly good quantitative agreement with the experimentally observed time courses (RMS error given as Supplementary Table S2). For the other cases, the kinetic model correctly predicts approximately no surface area change (no competition) when similar populations are mixed, or when a population is mixed with buffer.

Middle figures 4c and 4d show phospholipid-driven competition from a different angle: that of *vesicle stoichiometry*. Stoichiometry explores the final equilibrium size of vesicles in a tracked population, when this population is mixed with a different population containing approximately *R* times as many vesicles. In this approach, the trend of final equilibrium surface area size versus mixing ratio is explored, rather than the dynamics on the way to equilibrium. Figure 4c details final surface area of a tracked population of DOPA:OA (*ρ*_0_ = 0.1) vesicles, when this population is mixed 1:*R* with a population of pure OA vesicles. Figure 4d details the opposite scenario, whereby the tracked population is OA vesicles, mixed 1:*R* with DOPA:OA vesicles. The *R* = 1 cases in Figs 4c and 4d correspond to the surface sizes reached in the limit of time in Figs 4a and 4b, respectively.

Calculating competition equilibrium by means of the fast computation approach outlined in the Methods section, we were able to verify that our model exhibits continual growth of DOPA:OA (*ρ*_0_ = 0.1) vesicles as more OA vesicles are added (Fig. 4c). If vesicles started at 5% deflation, the model matched the experimental data points even more closely. In the opposite scenario, we also verified that the model shows the same distinctive plateau in the shrinkage of pure OA vesicles as more DOPA:OA (*ρ*_0_ = 0.1) vesicles are added (Fig. 4d). In the case of the latter figure, notably the *indirect effect* alone is sufficient to reproduce experimental results.

Importantly, the general outcome of phospholipid-driven competition in our model is for DOPA:OA mixed vesicles to uptake lipid, grow in surface and to finish at high Φ > 1 values (excess surface, flaccid), whereas pure OA vesicles lose lipid, suffer reduced surface, and finish at Φ < 1 values (osmotically tense, spherical). This is observed experimentally, and indeed provides the basis for the conjecture that phospholipid-laden vesicles are more likely to divide spontaneously when gentle external shearing forces are applied^17^.

Moving to osmotically-driven competition, Fig. 4e shows simulation of a swelled population of vesicles competing with an initially isotonic (non-swelled) population. Simulation outcomes match quite well the experimental best-fit time courses, in particular for the growth of the swelled vesicles (less accurately for the shrinkage of the non-swelled vesicles). In any case, it must be noted that the original experimental data (yellow data points) has considerable variance. Then, Fig. 4f shows that the kinetics model qualitatively reproduces the stoichiometric observation whereby adding more swelled vesicles to a population of initially non-swelled vesicles will cause the shrinkage of the non-swelled vesicles to plateau, rather than to continue (note the logarithmic scale of Fig. 4f). Again, model outcomes are improved if vesicles start at 5% deflation.

The general outcome of osmotically-driven competition in our model is for all vesicles to finish with different surface sizes (as for phospholipid-driven competition), but now, all vesicles also share the same Φ < 1 value, indicating equal osmotic stress. This residual osmotic stress is also observed experimentally and stands as the main criticism of the osmotically-driven competition scenario. In order to divide, swelled vesicles would have to overcome a stronger energetic barrier, changing their stressed membrane state into one ready for fission, making this an improbable route to spontaneous vesicle reproduction^18^.

**Figure 5:**
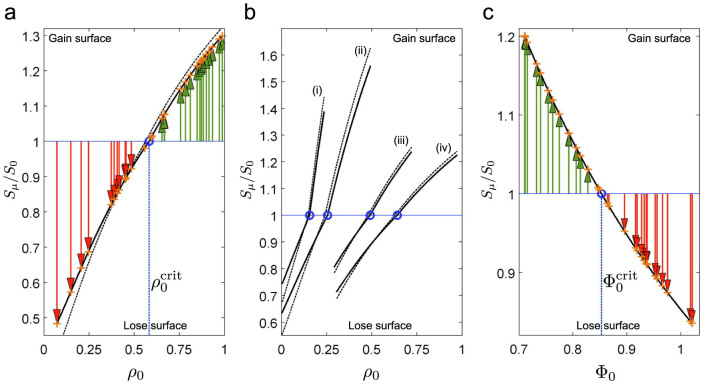
Lipid competition tipping points. (a) Phospholipid competition between 30 model phospholipid-laden vesicles each with a different DOPA fraction randomly assigned over the uniform interval 0 < *ρ*_0_ < 1 and initially spherical, 120 nm diameter. Depending on initial DOPA fraction, each vesicle starts at a point on the horizontal blue line, and grows (green arrows) or shrinks (red arrows) to a point on the black line. The form of the black line is *specific* to this particular competing population, and is computed by equation (15). Competition ‘tipping point’ is shown by blue circle: any vesicle with 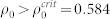 gains lipids from its competitors. The solid black line shows relative growths when only the indirect effect exists (*d* = 0); for comparison, the dashed black line shows relative growths when the direct effect also maximally present (*d* = 1). Initial fatty acid concentration inside and outside model vesicles was [*L*] = 5.0 × 10^−5^ *M*. (b) Phospholipid competition in four unique populations of 30 model vesicles, with DOPA fraction randomly assigned over uniform intervals (i) 0 < *ρ*_0_ < 0.25, (ii) 0 < *ρ*_0_ < 0.5, (iii) 0.25 < *ρ*_0_ < 0.75 and (iv) 0.3 < *ρ*_0_ < 1.0, demonstrating the context-dependence of competition. (c) Osmotic competition between 30 model oleate vesicles, each with 120 nm surface diameter and each starting with a different degree of swelling, from maximally swelled to 5% deflated (Φ_0_ randomly assigned over uniform interval 0.7 < Φ_0_ < 1.0348). Any vesicle starting at tension state 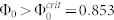 gains lipids from its competitors. Initial fatty acid concentration inside and outside vesicles was [*L*] = 6.67 × 10^−5^ *M*. Environment volume for competition in all three plots was 1.04 × 10^−14^ litres. Orange crosses show agreement with equilibrated deterministic simulation of the model.

**Figure 6:**
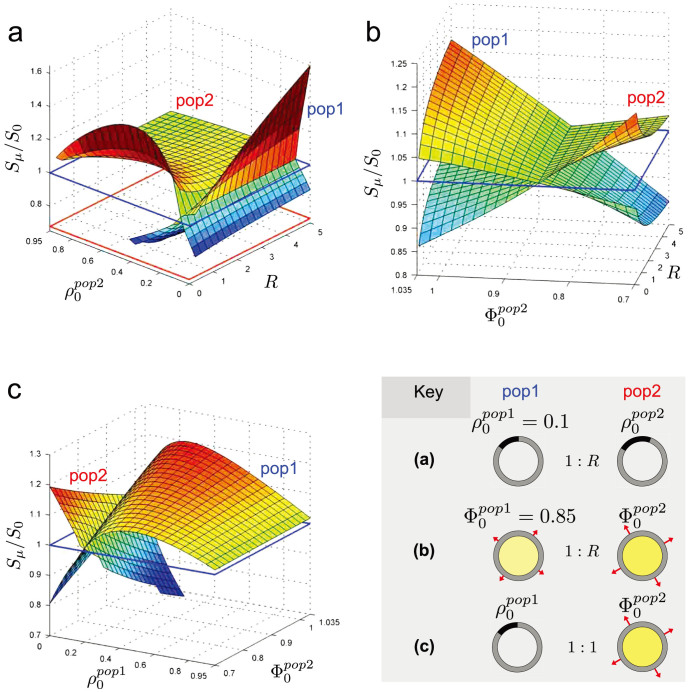
Wider exploration of three different vesicle competition scenarios. Relative surface growths of two vesicle populations is explored in a broader context for three different competition scenarios detailed by the key. All model vesicles are considered to be 120 nm diameter. Additionally, the DOPA:OA vesicles are considered 5% deflated upon extrusion, and only have the indirect effect present (*d* = 0). (a) Phospholipid-driven competition. Population 1, a fixed population of vesicles with initial DOPA phospholipid fraction 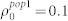, is mixed 1:*R* with population 2, whose vesicles have initial DOPA fraction 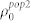. (b) Osmotically-driven competition. Population 1, a fixed population of initially swelled 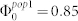 vesicles, is mixed 1:*R* with population 2, whose vesicles are initially swelled by 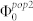. (c) Phospholipid versus osmotically-driven competition. Vesicles with initial DOPA fraction 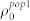 are mixed 1:1 with pure oleate vesicles swelled by 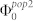. Blue box outlines on the 3d plots highlight when the relative surface growth is 1.

Our kinetic model can also be used to make predictions or to find competition ‘tipping points’ in the more general scenario where completely heterogeneous populations of phospholipid-laden and/or osmotically swollen vesicles compete for lipid (Figs. 5 and 6), even if some of these experiments have not been realised in the lab yet.

### Competition tipping points in diverse populations

Figure 5a shows that within a hypothetical population of model phospholipid-laden vesicles, where each vesicle has a randomly assigned phospholipid fraction in the membrane between 0 and 100%, the critical DOPA fraction needed for growth (tipping point), in this case, is just over 58%.

Figure 5b compares different heterogeneous populations competing for phospholipid, and reveals an important observation: *competition is always context dependent*. That is to say, a certain amount of membrane phospholipid does not guarantee a certain final surface area. Rather, final surface depends on the boundary conditions of the competition event (that is, the parameters influencing the solution of equation (15) in the later Methods section), which includes the number and composition of competitor vesicles present. For example, population (i) in Fig. 5b has vesicles with low DOPA fraction as compared to vesicles in population (iv), yet in some cases the vesicles in the former population have larger final surface growth than vesicles in the latter. This concurs with the experimental observation that even small differences in phospholipid content should drive growth^17^.

The dotted black lines in Figs 5a and 5b are the same competition events run when the *direct effect* is present, and maximally enabled (*d* = 1). The extent to which the direct effect affects vesicle growth must be made on a case by case basis, as it depends on the specifics of the competition event. For example, the direct effect has marginal influence on vesicle growth trends in the population shown in Fig. 5b (iii), but is more relevant in population (ii). The Supplementary material contains a recalculation of both Fig. 5a and 5b, if we further take into account the realistic constraint that vesicles will burst when osmotic tension exceeds a critical limit (Φ < 0.7).

Figure 5c shows that in a heterogeneous population where pure OA model vesicles are swelled to differing extents, vesicles with low initial Φ values take lipid from those with higher (less swelled) Φ values, with the tipping point between growing and shrinking at 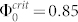. As a last remark, orange crosses marked on Figs 5a and 5c show that full deterministic simulations of the model (run all the way to equilibrium) agree with and thus validate the ‘Fast Computation of Competition Equilibrium’ procedure outlined in the Methods section.

### Theoretical predictions beyond current experimental results

Finally, we were able to explore more widely some of the parameter space for phospholipid-driven and osmotically-driven competition, using our model to make some predictions. Figure 6a shows the stoichiometry results of phospholipid-driven competition in this wider context. A population of DOPA:OA (*ρ*_0_ = 0.1) vesicles is mixed with a second population, but the phospholipid content of the second population, as well as the mixing ratio *R*, are varied. Taking a slice through the surface labelled ‘pop1’ when 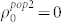 shows the result reported as the solid red line in Fig. 4c. Figure 6b explores the stoichiometry of osmotically-driven competition in a similar way to phospholipid-driven competition. A fixed population of swelled vesicles is mixed with a second population, where the degree of swelling in the second population, as well as the mixing ratio *R* are varied. To conclude these predictions, Fig. 6c shows the effects of osmotically-driven versus phospholipid-driven competition, still a completely unreported scenario in the experimental literature, whereby a population of swelled pure oleate vesicles competes for lipid with a population of DOPA:OA vesicles. The swelled oleate vesicles are able to steal lipid from the DOPA:OA vesicles, when the former have a high degree of swelling and the latter have a low DOPA fraction; otherwise, the DOPA:OA vesicles prosper in the competition.

Vesicle bursting is an important consideration in Fig. 6. Competition predictions in Fig. 6a are only strictly valid when the population 1 surface is above the red box lines. Below these lines, vesicles in population 1 have excessive osmotic pressure (Φ < 0.7) and would likely burst, altering competition outcomes for population 2. Likewise, the population 2 surface in Fig. 6c is only drawn for values where oleate vesicles in that population have final Φ > 0.7. Outside the extent of the population 2 surface, competition outcomes for population 1 should be treated with caution, as not all population 2 vesicles will be intact.

## Discussion

In this work we have presented a theoretical model of the transfer kinetics of single chain fatty acids between competing vesicles. We have shown that data coming from controlled laboratory experiments on phospholipid-driven competition and osmotically-driven competition can be reproduced fairly well by a set of physically-based rate equations describing the uptake and release of fatty acids for each vesicle. Furthermore, we have been able to predict the outcome of several yet-to-be-performed experiments. Thus, it is time to recapitulate, considering possible limitations of our approach, clarifying several points that remain open, and giving a more general perspective on the problem addressed.

The main assumption we made when modelling phospholipid-driven competition is that the phospholipids are not released by vesicle membranes at the timescale of fatty acid transfer between the supramolecular structure (i.e., the closed membrane bilayer) and the aqueous solution (both inwards and outwards). This assumption is well founded on experimental evidence^14^,^15^ and a previous theoretical model^16^. Likewise, the assumption we make with osmotically-driven competition is that the extra buffer present inside the vesicles, swelling them, permeates very slowly through the bilayer membrane. In reality, the off-rate of a lipid molecule from a bilayer is inversely correlated with the number of carbon atoms in the acyl chain of the lipid concerned, and phospholipids do have a small non-zero transfer rate (with a half time from hours to days^16^,^17^). If the much slower phospholipid transfer was included in our model, the equilibrium reached in the limit of time would always be that of a completely homogeneous population. This is because the *P* phospholipid would redistribute amongst the vesicles until all were equilibrated with the same phospholipid monomer concentration in solution [*P*]*_eq_*, which is trivially when all vesicles have the same fraction of membrane phospholipids *P_μ_*. With no remaining asymmetries in *P_μ_* fraction to drive competition, all vesicles would finish with the same lipid composition and same surface size. The initial appearance and eventual disappearance of competition would thus follow the same type of dynamics as those experimentally reported by Budin & Szostak^17^ (Fig. 3D therein) for nervonic acid, which redistributes between vesicles. If vesicles contained a metabolism which synthesised phospholipid, then lasting *P_μ_* asymmetries between vesicles could conceivably be maintained as steady states, despite a continuous process of *P* exchange. However, in this work we took the route of *not* explicitly modelling phospholipid synthesis, to reduce the competition scenario to a materially-closed system which subsequently settles to equilibrium. Under this condition, analysis is easier to perform. In summary, the results of this study can be interpreted as reflecting the competition advantage bestowed upon a vesicle by a membrane phospholipid fraction *given that this fraction is somehow maintained as constant*.

The next point that deserves discussion is the the causative role of the *direct effect* in the phospholipid-driven competition simulations performed with our model. Our choice for function **r** means that a DOPA:OA vesicle with 10 mol% DOPA fraction will have fatty acids leaving the bilayer at reduced rate **r**(0.1) × *k_out_* = 0.9*k_out_* when the direct effect is maximal (*d* = 1). It could be argued that other function choices for **r** could reduce fatty acid off-rate even further for the same DOPA fraction. However, a large direct effect is not needed to best fit model outcomes with experimental outcomes, and would actually make the fit worse. Examination of Figs 4b and 4d in fact shows that having only the indirect effect provides the best fit to experimental outcomes (quantified in Supplementary Table S2). On the other hand, the dynamics and stoichiometry outcomes of Figs 4a and 4c respectively are only improved when there is a small reduction of *k_out_*: a small direct effect of around 0 < *d* < 1 for Fig. 4a and with *d* a little larger than 1 for Fig. 4c. With the maximal level of direct effect provided by our function **r**, Supplementary Table S3 shows that the direct effect only accounts for around 20% of the total vesicle surface growth. Therefore, we should conclude that in our kinetic treatment of vesicle competition, the indirect effect is the main mechanism driving vesicle growth dynamics.

One curiosity in the results (both *in vitro* and *in silico*) is how DOPA:OA (*ρ*_0_ = 0.1) vesicles grow continually as more OA vesicles are added (Fig. 4c). This is unintuitive, since the growth of the DOPA:OA vesicles should imply a dilution of their phospholipid content, which would seemingly reduce the indirect and direct effects, thus giving a negative feedback to eventually curb the DOPA:OA growth profile. The reason why our model reproduces this continuous growth result has to do with the mathematics underlying the kinetic modelling. In the limit of infinite *L_μ_* lipids in the membranes of our model DOPA:OA vesicles, the inside/outside lipid concentration required to sustain them at equilibrium (given by function *f*, as defined by equation (13) later in the Methods section) tends to, *but crucially never actually reaches*, the CVC of pure oleic acid: 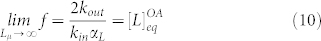
This is true, even if a model DOPA:OA vesicle contains just one single phospholipid in the membrane. Now, as more OA vesicles are mixed with the DOPA:OA vesicles, the population becomes increasingly dominated by OA vesicles and the lipid monomer concentration in the environment subsequently rises toward 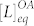. As this happens, equation (10) implies that the DOPA:OA vesicles will be absorbing more and more *L* lipids, in order to grow to a size that is in equilibrium with the external lipid monomer concentration. The growth of our model DOPA:OA vesicles is thus halted only by the number of lipids in the system being limited to *L_t_* and not by dilution of the membrane phospholipid fraction. In our kinetics model, continuous growth happens with or without the direct effect present.

A final point worth highlighting is that when the lipid uptake function **u** given in equation (2) is not conditional, as we assumed, but simply 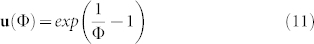
for all membrane states (which denotes that even flaccid vesicles have differential rates of lipid uptake), then, quite interestingly, the continuous DOPA:OA growth effect cannot be reproduced. If we use definition (11) for function **u** in function *f* (13), and call the new function *f_nc_*, it can be shown that 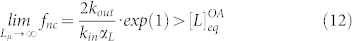
meaning that the DOPA:OA vesicles do not show the same continued growth as the lipid monomer concentration in the environment rises toward 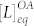. Rather, the DOPA:OA have much slower growth, and they even have a finite stable size when the outside lipid monomer concentration is exactly 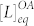. Thus, to best reproduce experimental outcomes, a crucial part of our lipid uptake kinetics was to accelerate lipid uptake *only* in osmotically stressed vesicle states, not in flaccid ones. This is a new addition to our general kinetic model, introduced in this paper.

This work is a step forward in the development of semi-realistic, coarse-grained descriptions of phenomena that, in reality, are extremely complex. Self-assembly processes involving heterogeneous component mixtures and the formation of dynamic supramolecular structures that could hypothetically lead to biologically relevant forms of material organization, like protocells^28^,^29^, constitute a tremendous challenge, indeed, both for experimental and theoretical ‘systems chemistry’ research^30^ and for synthetic biology^7^,^31^. In particular, the connection between basic metabolic reaction networks and membrane dynamics (including stationary growth and division cycles^32^) needs to be explored much more extensively, since it is one of the key aspects to establish a plausible route from physics and chemistry towards biological phenomenology.

## Methods

### Fast computation of competition equilibrium

Here we provide a general numerical approach to solving the equilibrium configuration of a possibly heterogeneous population of vesicles competing for a limited supply of lipid. These vesicles may be osmotically swelled, laden with phospholipid, or a mixture of both, and can be arbitrary in number. The method allows the lipid uptake and release functions **u** and **r** to take arbitrary forms, subject to some requirements detailed below.

We start by defining a function *f*:*L_μ_* → [*L*]*_eq_*, like equation (7), which gives the inside/outside lipid monomer concentration [*L*]*_eq_* necessary to maintain a particular vesicle 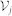 at equilibrium, given that this vesicle has a specific number of lipids/phospholipids in the membrane, and a specific volume: 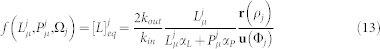
The inverse of this function yields useful information: it is the mapping of [*L*]*_eq_* to the number of lipids which must exist in the membrane of a particular vesicle, in order for that vesicle to be at equilibrium.

However, due to the difficulty in isolating *L_μ_* from the potentially non-linear functions **u** and **r**, in most cases the inverse mapping is not possible to write in closed form. Nevertheless, if uptake and release functions **u** and **r** make function *f* (i) one-to-one, (ii) onto and (iii) continuous, then it follows that the inverse mapping is a function *f*^−1^, which can be numerically calculated for vesicle 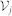 by using *f* and binary searching for an *L_μ_* which satisfies: 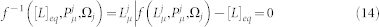
using appropriate search bounds (normally: 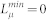, 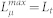).

Crucially, having a means to calculate *f*^−1^ gives a way of determining the total number of lipids existing in all equilibrated vesicle membranes, given that the inside/outside lipid monomer concentration in the heterogeneous vesicle mixture is [*L*]*_eq_*. For each [*L*]*_eq_*, we know that each vesicle has a *unique* number of membrane lipids *L_μ_*, because *f*^−1^ is itself one-to-one. This means that a certain [*L*]*_eq_* can only admit one single equilibrium configuration of vesicles, not multiple equilibrium configurations, and this lack of ambiguity is a desirable property for the method.

The lipid monomer concentration [*L*]* inside/outside all vesicles in this single equilibrium configuration can be found by making use of the lipid conservation principle in equation (5): 
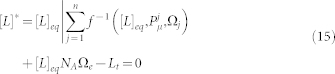
That is, at [*L*]*, the lipid making up the membranes of all equilibrated vesicles, plus the lipid monomer inside and outside the vesicles is equal to the total lipid in the system *L_t_* set by the initial condition. Expression (15) can also be solved by binary search of [*L*]*_eq_* between appropriate bounds, normally 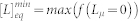 over all vesicles, [*L*]*_eq_*^*max*^ = *min*(*f*(*L_μ_* = *L_t_*)) over all vesicles. Finally, knowing [*L*]* allows to fully reconstruct the final sizes of all vesicles at equilibrium by substituting [*L*]*_eq_* = [*L*]* into equation (14) for each vesicle 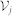.

In the equilibrated population, some vesicles will have grown larger in surface area at the expense of others which will have shrunk. When a population of vesicles has competed for lipid via phospholipid-driven competition, the ‘tipping point’ is the critical number of membrane phospholipids 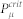 separating those vesicles which have lost lipid from those which have gained lipid, and is found by: 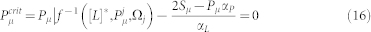
where 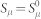, again solvable by binary searching, this time in the range 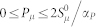, (from a pure lipid membrane to a pure phospholipid membrane). Expression (16) amounts to asking how many phospholipids a hypothetical vesicle would require in order not to grow in surface area when the lipid monomer concentration has stabilised at [*L*]*. Likewise, the number of phospholipids required to achieve any arbitrary surface area growth can be found by setting *S_μ_* to the value desired.

The critical phospholipid number can be stated more usefully as the critical phospholipid molecular fraction 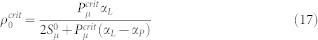
a vesicle has in the initial condition, a time when all vesicles have a surface of 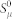. For osmotically-driven competition, the critical volume separating shrinking vesicles from growing vesicles is found by searching expression (16) for vesicle volume instead: 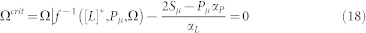
where 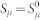. This may be alternatively stated as the critical Φ in the initial condition: 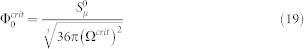
If no sign change results when evaluating the functions given in equations (14–18) at the upper and lower search bounds, then the respective equation cannot be solved by this numerical bisection approach. Otherwise typically 30 iterations of binary search were used to converge to an accurate answer.

## Author Contributions

All authors helped conceive the model. B.S.-E. and R.S. analysed the model, and B.S.-E. conducted the numeric simulations. B.S.-E., K.R.-M. and R.S. wrote the paper. F.M. provided crucial feedback on several aspects.

## Supplementary Material

Supplementary Information Supplementary Material for: Modelling Lipid Competition Dynamics in Heterogeneous Protocell Populations

## Figures and Tables

**Figure 1:**
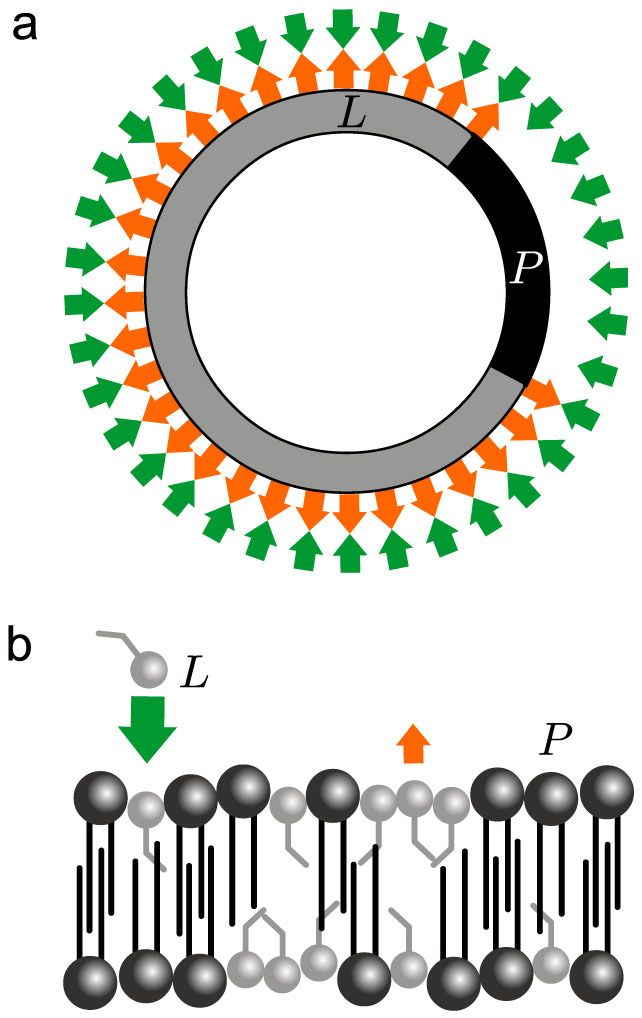
Two mechanisms of phospholipid-driven growth. (a) *Indirect effect*, whereby the presence of phospholipid in a vesicle membrane drives growth simply through a geometric asymmetry: only the lipid section of the bilayer (grey) is able to release lipid (orange arrows) whereas the whole of the bilayer surface (made of lipids and phospholipids) is able to absorb lipid monomer (green arrows). Phospholipid fraction is pictured as one continuous block to highlight the principle only. The indirect effect can be created also by non-lipid surfactant molecules (e.g. peptides) residing long enough in the membrane to increase surface absorption area. (b) *Direct effect*, whereby the acyl tails of the phospholipids have high affinity for packing closer to each other and increasing bilayer order, thus making the exit of the simple lipids more difficult. The direct effect is specific to the molecular structure of phospholipids.

**Table 1:** Vesicle competition model parameters. ^(*a*,*b*)^Area and volume of 120 nm diameter sphere. ^(*c*)^ 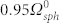 reported in^24^. ^(*d*)^ Close to 80 *μM* value reported for oleate vesicles^15^. ^(*e*)^ Bicine buffer concentration used experimentally^15^,^17^. ^(*f*)^ See Supplementary Material for rationale. ^(*g*)^ Reported in^33^. ^(*h*)^ Considered similar to POPC head area reported in^33^. ^(*i*)^ Calculated from equation (13); same for spherical and deflated vesicles. ^(*j*)^ Calculated as: 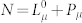 where 
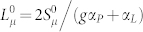
, 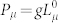 and 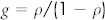

Parameter	Description	Value	Unit
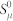	Inside/ouside surface area of extruded model vesicles^(*a*)^	4.524 × 10^4^	*nm*^2^
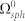	Volume of model vesicles, spherical when extruded^(*b*)^	9.048 × 10^5^	*nm*^3^
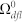	Volume of model vesicles, deflated when extruded^(*c*)^	8.595 × 10^5^	*nm*^3^
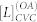	Critical vesicle concentration for oleic acid^(*d*)^	6.667 × 10^−5^	*M*
[*B*]	Buffer concentration^(*e*)^	0.2	*M*
*k_out_*	OA lipid release constant^(*f*)^	7.6 × 10^−2^	*s*^−1^
*k_in_*	OA lipid uptake constant^(*f*)^	7.6 × 10^3^	*s*^−1^*M*^−1^*nm*^−2^
*α_L_*	OA lipid head area^(*g*)^	0.3	*nm*^2^
*α_P_*	DOPA lipid head area^(*h*)^	0.7	*nm*^2^
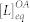	OA monomer equilibrium concentration, for pure OA vesicle^(*i*)^	6.667 × 10^−5^	*M*
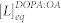	OA monomer equilibrium concentration, for DOPA:OA (*ρ*_0_ = 0.1, *d* = 0) vesicle^(*i*)^	5.297 × 10^−5^	*M*
*N^OA^*	Aggregation number for extruded pure OA vesicle^(*j*)^	301592	
*N^DOPA:OA^*	Aggregation number for extruded DOPA:OA (*ρ*_0_ = 0.1, *d* = 0) vesicle^(*j*)^	266111	

## References

[b1] Smith J. M. & Szathmáry E. The Major Transitions in Evolution (Oxford University Press, 1995).

[b2] Luisi P. L. The Emergence of Life: From Chemical Origins to Synthetic Biology (Cambridge University Press, 2006).

[b3] Varela F. G., Maturana H. R. & Uribe R. Autopoiesis: the organization of living systems, its characterization and a model. Biosystems 5, 187–196 (1974). 10.1016/0303-2647(74)90031-8 4407425

[b4] Gánti T. Organization of chemical reactions into dividing and metabolising units: the chemotons. Biosystems 7, 15–21 (1975). 1156666 10.1016/0303-2647(75)90038-6

[b5] Dyson F. Origins of Life (Cambridge University Press, 1985).

[b6] Segré D., Ben-Eli D., Deamer D. W. & Lancet D. The lipid world. Orig Life Evol Biosph 31, 119–145 (2001). 11296516 10.1023/a:1006746807104

[b7] Solé R. V., Munteanu A., Rodriguez-Caso C. & Macía J. Synthetic protocell biology: from reproduction to computation. Philos Trans R Soc Lond B Biol Sci 362, 1727–1739 (2007). 17472932 10.1098/rstb.2007.2065 PMC2442389

[b8] Macía J. & Solé R. V. Synthetic turing protocells: vesicle self-reproduction through symmetry-breaking instabilities. Philos Trans R Soc Lond B Biol Sci 362, 1821–1829 (2007). 17510018 10.1098/rstb.2007.2074 PMC2442396

[b9] Mavelli F. & Ruiz-Mirazo K. Stochastic simulations of minimal self-reproducing cellular systems. Philos Trans R Soc Lond B Biol Sci 362, 1789–1802 (2007). 17510021 10.1098/rstb.2007.2071 PMC2515193

[b10] Ruiz-Mirazo K., Piedrafita G., Ciriaco F. & Mavelli F. Stochastic Simulations of Mixed-Lipid Compartments: From Self-Assembling Vesicles to Self-Producing Protocells. vol. 696 of Adv Exp Med Biol, 689–696 (Springer, 2011). 10.1007/978-1-4419-7046-6_70 21431610

[b11] Mavelli F. Stochastic simulations of minimal cells: the ribocell model. BMC Bioinformatics 13, S10 (2012). 22536956 10.1186/1471-2105-13-S4-S10 PMC3303737

[b12] Munteanu A., Attolini C. S.-O., Rasmussen S., Ziock H. & Solé R. V. Generic darwinian selection in catalytic protocell assemblies. Philos Trans R Soc Lond B Biol Sci 362, 1847–1855 (2007). 17510015 10.1098/rstb.2007.2077 PMC2442399

[b13] Mavelli F. & Stano P. Kinetic models for autopoietic chemical systems: the role of fluctuations in a homeostatic regime. Phys Biol 7, 16010 (2010). 20130341 10.1088/1478-3975/7/1/016010

[b14] Cheng Z. & Luisi P. L. Coexistence and mutual competition of vesicles with different size distributions. J Phys Chem B 107, 10940–10945 (2003).

[b15] Chen I. A., Roberts R. & Szostak J. W. The emergence of competition between model protocells. Science 305, 1474–1476 (2004). 15353806 10.1126/science.1100757 PMC4484590

[b16] Mavelli F. & Ruiz-Mirazo K. Environment: a computational platform to stochastically simulate reacting and self-reproducing lipid compartments. Phys Biol 7, 036002 (2010). 20702920 10.1088/1478-3975/7/3/036002

[b17] Budin I. & Szostak J. Physical effects underlying the transition from primitive to modern cell membranes. Proc Natl Acad Sci U S A 108, 5249–5254 (2011). 21402937 10.1073/pnas.1100498108 PMC3069173

[b18] Adamala K. & Szostak J. W. Competition between model protocells driven by an encapsulated catalyst. Nat Chem 5, 495–501 (2013). 23695631 10.1038/nchem.1650 PMC4041014

[b19] Erdi P. & Tóth J. Mathematical models of chemical reactions: Theory and applications of deterministic and stochastic models (Manchester University Press, 1989).

[b20] Ullah M. & Wolkenhauer O. Stochastic Approaches for Systems Biology (Springer, 2011). 10.1002/wsbm.78 20836037

[b21] Simard J. R., Pillai B. K. & Hamilton J. A. Fatty acid flip-flop in a model membrane is faster than desorption into the aqueous phase. Biochemistry 47, 9081–9089 (2008). 18693753 10.1021/bi800697q

[b22] Kamp F. & Hamilton J. A. ph gradients across phospholipid membranes caused by fast flip-flop of un-ionized fatty acids. Proc Natl Acad Sci U S A 89, 11367–11370 (1992). 1454821 10.1073/pnas.89.23.11367 PMC50551

[b23] Chen I. A. & Szostak J. W. Membrane growth can generate a transmembrane ph gradient in fatty acid vesicles. Proc Natl Acad Sci U S A 101, 7965–7970 (2004). 15148394 10.1073/pnas.0308045101 PMC419540

[b24] Mally M., Peterlin P. & Svetina S. Partitioning of oleic acid into phosphatidylcholine membranes is amplified by strain. J Phys Chem B 117, 12086–12094 (2013). 24000876 10.1021/jp404135g

[b25] Cape J. L., Monnard P.-A. & Boncella J. M. Prebiotically relevant mixed fatty acid vesicles support anionic solute encapsulation and photochemically catalyzed trans-membrane charge transport. Chem Sci 2, 661–671 (2011).

[b26] Lotka A. J. Elements of Physical Biology (Williams and Wilkins, Baltimore, 1925).

[b27] Mui B. L.-S., Cullis P. R., Evans E. A. & Madden T. D. Osmotic properties of large unilamellar vesicles prepared by extrusion. Biophys J 64, 443–453 (1993). 8457670 10.1016/S0006-3495(93)81385-7 PMC1262347

[b28] Mouritsen O. G. Life - As a Matter of Fat: The Emerging Science of Lipidomics (Springer-Verlag, 2005).

[b29] Rasmussen, S. *et al.* (eds.) Protocells: Bridging Non-Living and Living Matter (MIT Press, 2009).

[b30] Ruiz-Mirazo K., Briones C. & de la Escosura A. Prebiotic systems chemistry: New perspectives for the origins of life. Chem Rev 114, 285–366 (2014). 24171674 10.1021/cr2004844

[b31] Solé R. V. Evolution and self-assembly of protocells. Int J Biochem Cell Biol 41, 274–284 (2009). 18951997 10.1016/j.biocel.2008.10.004

[b32] Mavelli F. & Ruiz-Mirazo K. Theoretical conditions for the stationary reproduction of model protocells. Integr Biol 5, 324–341 (2013). 10.1039/c2ib20222k 23233152

[b33] Lonchin S., Luisi P. L., Walde P. & Robinson B. H. A matrix effect in mixed phospholipid/fatty acid vesicle formation. J Phys Chem B 103, 10910–10916 (1999).

