# Supplementary material for: Modelling Lipid Competition Dynamics in Heterogeneous Protocell Populations
Source: Sci Rep. 2014 Jul 14;4:5675. doi: 10.1038/srep05675 (PMC4097352; doi:10.1038/srep05675)
Supplement: Supplementary Information — Supplementary Material for: Modelling Lipid Competition Dynamics in Heterogeneous Protocell Populations [file srep05675-s1.pdf]

Supplementary Material for:
Modelling Lipid Competition Dynamics in Heterogeneous Protocell Populations

Ben Shirt-Ediss[1,2], Kepa Ruiz-Mirazo[3,2], Fabio Mavelli[4], and Ricard V. Solé[1,5*]

E-mail: ricard.sole@upf.edu

Contents

1. Lipid Uptake Function **S2**

2. Fatty Acid Uptake and Release Kinetic Constants **S3**

2.1 Vesicle Equilibrium at CVC S3

2.2 Fit to Experimental Time Courses for Osmotic Competition S4

3. Modelling Vesicle Mixing **S4**

3.1 Competition Volume S5

3.2 Mixing for Phospholipid-Driven Competition S6

3.3 Mixing for Osmotically-Driven Competition S7

3.4 Control Experiments: Mixing with Buffer S9

3.5 Concentration Mixing vs. Volume Mixing S9

[*]To whom correspondence should be addressed
[1] ICREA-Complex Systems Lab, Institut de Biologia Evolutiva, CSIC-UPF, Barcelona, Spain
[2] Logic and Philosophy of Science Department, University of The Basque Country, Spain
[3] Biophysics Unit (CSIC-UPV/EHU), University of The Basque Country, Spain
[4] Chemistry Department, University of Bari, Italy
[5] Santa Fe Institute, 1399 Hyde Park Road, Santa Fe NM 87501, USA

4. **Quantitative Fit Between Model Predictions and Experimental Outcomes** **S10**

5. **Vesicle Breakage** **S12**

1. Lipid Uptake Function

The exponential function is derived from the Arrenhius equation that describes the dependence of a kinetic constant k on the absolute temperature T and on the activation energy E_a:

$$k = A \cdot exp\left(-\frac{E_a}{k_B T}\right) \tag{1}$$

where the pre-exponential factor A is considered constant. By assuming that the activation energy linearly decreases when the membrane is stretched (for instance, due to osmotic stress), inducing an increase in its surface area ($\Delta S > 0$), E_a can be expressed as a function of Φ:

$$E_a(\Phi) = E_a^{\oslash} - \Delta E_a\left(\frac{\Delta S}{S^{\oslash}}\right) = E_a^{\oslash} - \Delta E_a\left(\frac{1}{\Phi} - 1\right) \tag{2}$$

where $E_a^{\oslash}$ is the activation energy for a spherical vesicle membrane of surface $S^{\oslash}$, while $-\Delta E_a(\Delta S/S^{\oslash})$ represents the activation energy reduction due to the surface increase ΔS. Therefore, if $\Delta E_a \approx k_B T$, $k_{in}(\Phi)$ can be expressed in terms of the kinetic constant of spherical vesicles:

$$k_{in}(\Phi) \approx k_{in}^{\oslash} \cdot exp\left(\frac{1}{\Phi} - 1\right) \tag{3}$$

$\Delta E_a \approx \gamma_{OA}\alpha_{OA}$ can be estimated as the work to be done in order to proportionally stretch the head area of each oleic acid molecule ($\alpha_{OA} = 0.3nm^2$, ref[1]), $\gamma_{OA} = 32.5$ dyne/cm being the surface tension of oleic acid at 298.15K (ref[2]), so that the ratio gives: $\gamma_{OA}\alpha_{OA}/k_B T = 2.37 \approx 1$ that could be a satisfying approximation.

2. Fatty Acid Uptake and Release Kinetic Constants

In this section, we give more detail on the two criteria used to set the k_{in} and k_{out} constants. These constants are derived from modelling pure fatty acid vesicles.

2.1 Vesicle Equilibrium at CVC

Criterion 1: *Pure fatty acid model vesicles, i.e. oleic acid vesicles, which are either spherical or deflated, must be in equilibrium and not changing in membrane size when the free fatty acid monomer in solution is the CVC for the fatty acid.*

When a model vesicle is in equilibrium, the rate of lipid uptake is equal to the rate of lipid release from the membrane:

$$k_{in} S_{\mu} [L]_{CVC} = k_{out} L_{\mu} \tag{4}$$

(Note that functions $\mathbf{u}$ and $\mathbf{r}$ have disappeared: for a pure fatty acid vesicle, $\rho = 0$ and thus $\mathbf{r}(\rho) = 1$. Further, when the vesicle is spherical or deflated, $\Phi \geq 1$ and so $\mathbf{u}(\rho) = 1$ also.)

Substituting surface area of the vesicle:

$$k_{in} \left(\frac{L_{\mu} \alpha_L}{2} \right) [L]_{CVC} = k_{out} L_{\mu} \tag{5}$$

Re-arranging:

$$\frac{2}{\alpha_L} \frac{k_{out}}{k_{in}} = [L]_{CVC}^{(OA)} \approx 70 \mu M \tag{6}$$

Criterion 1 enforces that k_{in} and k_{out} must be in a certain *ratio*. Increasing or decreasing the absolute magnitudes of k_{in}, k_{out} but maintaining the same ratio between them, increases or decreases, respectively, the velocity of the model dynamics in reaching competition equilibrium (but not the equilibrium point itself).

The second criterion below significantly narrows the possible pairs of k_{in}, k_{out}.

2.2 Fit to Experimental Time Courses for Osmotic Competition

Criterion 2: *Constants k_{in} and k_{out} must have absolute magnitudes such that the relative surface dynamics for a population of swelled OA vesicles competing 1:1 with a population of isotonic OA vesicles for the available lipid monomer, matches the best fit lines for the time courses experimentally reported by Chen et al.*[3] *from the perspectives of lowest RMS error and consistent shape.*

The Chen time course data for OA vesicle relative surface change as measured by FRET assay is best fitted by the following functions: $S_\mu/S_0 = 1.21 - 0.14e^{-0.08t}$ for swelled OA growth and $S_\mu/S_0 = 0.73 + 0.24e^{-0.09t}$ for isotonic OA shrinkage in 1:1 mixing.

Figure S1 shows that a good fit (low RMS error) between our model dynamics and the Chen experimental time course data is obtained when $k_{out} \approx 7.6 \times 10^{-2} s^{-1}$ (red line), which sets $k_{in} \approx 7.6 \times 10^{3} s^{-1} M^{-1} nm^{-2}$ accordingly. The general *shape* of the dynamics given by these values is also consistent with the Chen results.

3. Modelling Vesicle Mixing

This section defines how we model mixing two vesicle populations in our work, and thus how we interpret mix ratio R for vesicle stoichiometry. The mixing protocol, approximately based on a possible experimental procedure for mixing vesicles, allows us to initialise our theoretical model such that simulation results gained can be usefully compared against experimentally reported results.

More precisely, the mixing protocol establishes the following initial conditions for vesicle competition: (i) the number of vesicles present, (ii) their respective compositions, (iii) the environment volume Ω_e and (iv) the total amount of fatty acid L in the system (L_t). In what follows, simple lipids L are considered to be OA, and phospholipids P are considered

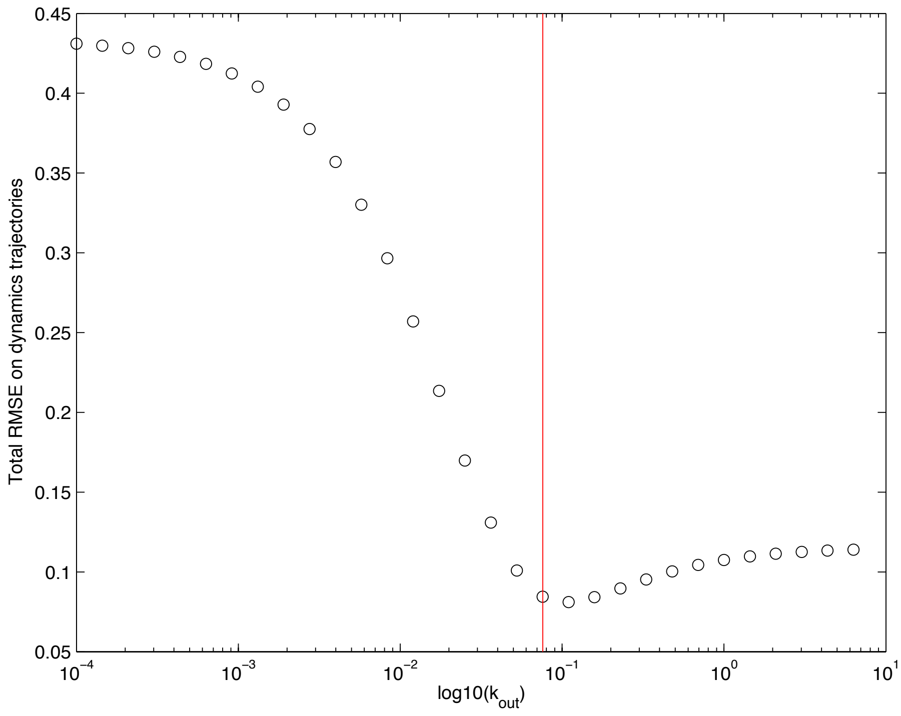

Figure S1: **Statistical comparison of RMS error between model and experimentally reported time series for osmotic competition as k_{out} is increased over 5 orders of magnitude.** RMS error reported is the total error in the model prediction summed over the swelled OA vesicle growth trajectory and the isotonic OA shrinkage trajectory. The red line marks $k_{out} \approx 7.6 \times 10^{-2} s^{-1}$ chosen for our model.

to be DOPA, to match experimental scenarios.

3.1 Competition Volume

The equilibrium finding method outlined in the 'Fast Computation of Competition Equilibrium' section of the paper requires summing over a finite number of vesicles. Likewise, dynamic simulations of the model require a finite ODE set. However, vesicle populations in a real laboratory experiment will typically have millions of vesicles competing for lipid. In our modelling approach, it is therefore necessary to consider a small volume 'patch' of each of the solutions being mixed. Each patch volume is large enough to contain enough vesicles so as to be *representative* of the vesicle density in the solution it pertains to, but no so many vesicles that numerical solution becomes infeasibly slow.

A patch volume $\Omega_p = \Omega_{stoi}$ litres (Supplementary Table S1) was utilised for stoichiometric calculations using the equilibrium finding method, which translates into around 2000 vesicles being involved in 1:1 mixing. Full dynamic simulation of the model with deterministic Runge-Kutta integration forced a yet smaller patch volume $\Omega_p = \Omega_{dyn}$ litres to be used, translating into around 40 vesicles being involved in 1:1 mixing.

3.2 Mixing for Phospholipid-Driven Competition

In order to mix a fixed population of DOPA:OA vesicles which have molecular fraction ρ of DOPA in their membranes, in ratio R with a variable population of pure OA vesicles, we assume the following basic steps.

Firstly, a suspension of OA lipid monomers in concentration RC_0 molar (assuming $RC_0 \gg CVC$ for oleic acid) is extruded (possibly multiple times) through 100nm diameter pores. We assume this leads to a more homogeneous population of 120nm diameter[1] pure OA unilamellar vesicles with surface S^0_μ. Vesicles are either assumed all spherical ($\Phi = 1$) with aqueous volume Ω^0_{sph}, or all deflated by 5% with aqueous volume Ω^0_{dfl} ($\Phi = 1.0348$). The molar concentration of OA vesicles in the extruded suspension is approximately

$$C^{OA}_{ves} = \frac{RC_0}{N^{OA}} \tag{7}$$

where N^{OA} is called the 'aggregation number', equal to the total number of lipids forming a vesicle bilayer (in this case, just OA lipids). The lipid monomer concentration in the aqueous solution inside/outside the vesicles is $[L]^{OA}_{eq}$, the CVC value, maintaining them at equilibrium (either in a spherical or deflated state).

Secondly, a mixed suspension containing both OA lipid monomers (in molar concentration C_0) and DOPA phospholipids (in molar concentration gC_0, where $g = \frac{\rho}{1-\rho}$) is extruded through 100nm diameter pores. This, similarly, leads to a population of 120nm diameter

[1] Choosing extrusion size to be 100nm or 110nm does not affect results. Vesicles have same *relative* surface growths in competition.

unilamellar DOPA:OA vesicles. Again, vesicles are either all spherical, or all slightly deflated by 5% as before, but now part of the bilayer consists of DOPA phospholipid in molecular fraction ρ. The molar concentration of DOPA:OA vesicles in the extruded suspension is approximately

$$C_{ves}^{DOPA:OA} = \frac{C_0(1+g)}{N^{DOPA:OA}} \tag{8}$$

where the aggregation number $N^{DOPA:OA}$ is now calculated as the sum of both the OA lipids and DOPA phospholipids making up each closed bilayer (see Table 1 of paper). In turn, the OA lipid monomer concentration inside/outside the vesicles is $[L]_{eq}^{DOPA:OA}$, the CVC value for the model DOPA:OA vesicles, maintaining them at equilibrium (equal for spherical or deflated DOPA:OA vesicles).

Competition starts ($t = 0$) when the extruded vesicle solutions above are mixed. We mix a volume Ω_p of each solution, creating a new mixed system of volume $\Omega_e = 2\Omega_p$, containing DOPA:OA vesicles in number $N_A \Omega_p C_{ves}^{DOPA:OA}$ and OA vesicles in number $N_A \Omega_p C_{ves}^{OA}$. The initial lipid monomer concentration in the environment becomes $\frac{1}{2}([L]_{eq}^{DOPA:OA} + [L]_{eq}^{OA})$. Throughout mixing, and during competition, buffer concentration is constant at $[B]$ in all solutions, at a value high enough for vesicles to maintain approximately constant volume.

Modelling the opposite scenario, namely a fixed population of pure OA vesicles mixed with a variable population of DOPA:OA vesicles, just requires switching the R multiplier from equation (7) to equation (8).

3.3 Mixing for Osmotically-Driven Competition

When a fixed population of isotonic OA vesicles is to be mixed in ratio R with a variable population of swelled OA vesicles, again two extruded vesicle suspensions are prepared. The first is prepared in buffer at molar concentration $[B]$ and extruded through 100nm diameter pores, leading to 120nm diameter unilamellar OA vesicles with surface S_μ^0, either

all spherical at volume Ω^0_{sph} ($\Phi = 1$), or all deflated by 5% at volume Ω^0_{dfl} ($\Phi = 1.0348$), in molar concentration

$$C^{isotonic}_{ves} = \frac{C_0}{N^{OA}} \tag{9}$$

The second suspension is prepared in a solution which contains an additional membrane impermeable (or slowly permeating) solute, such as sucrose, mixed with the buffer, increasing the overall molar concentration of osmotically active species to $[B]_0 \geq [B] + 0.7$. This suspension is made of unilamellar OA vesicles, either all spherical or all 5% deflated, in molar concentration $RC^{isotonic}_{ves}$. Each of these vesicles encapsulates buffer at high concentration $[B]_0$.

The buffer concentration outside the vesicles in the second suspension is then reduced to $[B]$, making the external solution hypotonic with respect to the vesicle interiors. The vesicles swell to maximum size[2], and then transiently break, allowing for the escape of buffer molecules in excess.

The vesicles later reseal with a residual buffer gradient of $[\Delta] = 0.16$M across the membrane, corresponding to a maximum osmotic pressure[3] of 4 atm. In our model, each vesicle, regardless if initially spherical or deflated, is therefore assumed to swell to volume $\Omega = \Omega^0_{sph}(1 + (0.16/[B]))$, which remains constant for the duration of competition.

The decrease of the environmental buffer concentration is considered to take place at the same instant of mixing with the initial isotonic population. This defines the initial condition ($t = 0$) when competition starts. The mixed overall volume is $\Omega_e = 2\Omega_p$ where isotonic vesicles number $N_A \Omega_p C^{isotonic}_{ves}$, and the swelled vesicles number an R multiple of this. The lipid monomer concentration in this new, larger environment is initially $[L]^{OA}_{eq}$.

[2]In the case of deflated vesicles, they first 'round up', before subsequently swelling to maximum size and breaking.

3.4 Control Experiments: Mixing with Buffer

Mixing a vesicle population with a buffer solution is modelled as doubling the current system volume and diluting the initial vesicle density to one half. In this case, we assume that the buffer solution contains no vesicles, but free lipid monomer at concentration just below the CVC of oleic acid, as is performed experimentally.

3.5 Concentration Mixing vs. Volume Mixing

The above procedures define a 'concentration approach' to mixing, where two equal volumes are mixed, and the number of vesicles in the variable population is controlled by increasing or decreasing vesicle concentration. Another approach to mixing would be the 'volume approach' whereby the variable population has a fixed vesicle concentration, but instead a variable volume which controls the number of vesicles present. Volume mixing was found to produce nearly equivalent outcomes in our model, so only results following the concentration mixing procedure are reported in the paper.

Table S1: **Parameters for vesicle mixing.**

Parameter	Description	Value	Unit
Ω_{stoi}	Competition volume unit for stoichiometric calculations	3.478×10^{-13}	dm^3
Ω_{dyn}	Competition volume unit for dynamics simulations	6.956×10^{-15}	dm^3
C_0	Mix concentration unit	0.001	M

4. Quantitative Fit Between Model Predictions and Experimental Outcomes

Supplementary Table S2 below uses RMS error to quantify how well model outcomes match those observed experimentally in Figure 4 of the paper. Supplementary Table S3 which then follows, calculates what percentage of vesicle surface change is attributable to the direct effect alone (when it is maximally present, $d = 1$) in Figures 4c and 4d of the paper.

Table S2: **RMS error of relative surface area prediction.** Data accompanies Figure 4 of paper to quantify how well model predicted outcomes match experimental results for vesicle surface dynamics and vesicle stoichiometry. Curve fitting for experimental time series data in Budin & Szostak[4] performed by 7th order polynomial, least squared error; experimental time series data in Chen et al.[3] fitted by exponential functions given in section 'Fatty Acid Uptake and Release Kinetic Constants' of this Supplementary Material. All DOPA:OA vesicles listed in the table have DOPA fraction of $\rho_0 = 0.1$.

			Spherical		*Deflated 5%*	
			$d = 0$	$d = 1$	$d = 0$	$d = 1$
Dynamics						
3a	DOPA:OA mixed 1:1 with	OA	0.0315	0.0140	0.0209	0.0238
		DOPA:OA	0.0145	0.0145	0.0145	0.0145
		BUFFER	0.0117	0.0159	0.0117	0.0159
3b	OA mixed 1:1 with	OA	0.0056	0.0056	0.0056	0.0056
		DOPA:OA	0.0153	0.0389	0.0161	0.0556
		BUFFER	0.0149	0.0149	0.0149	0.0149
3e	Swelled OA mixed 1:1 isotonic OA					
	Growth swelled		0.0119	0.0119	0.0175	0.0175
	Shrinkage isotonic		0.0817	0.0817	0.0670	0.0670
		TOTAL	0.1871	0.1974	0.1682	0.2148
Stoichiometry						
3c	DOPA:OA mixed 1:R with	OA	0.1897	0.1066	0.1392	0.0610
		DOPA:OA	0.0620	0.0620	0.0620	0.0620
3d	OA mixed 1:R with	OA	0.0215	0.0215	0.0215	0.0215
		DOPA:OA	0.0103	0.0299	0.0143	0.0503
3f			0.0875	0.0875	0.0711	0.0711
		TOTAL	0.3710	0.3075	0.3081	0.2659
	Dynamics + Stoichiometry	TOTAL	0.5581	0.5049	0.4763	0.4807

Table S3: **Importance of direct effect in driving surface change, for kinetic model.** Data reports vesicle stoichiometry data contained in Figure 4c and 4d of paper, for mixing unlike populations. The percentage overall surface change caused by the direct effect is calculated as the surface change caused only by the direct effect, divided by the total surface change caused by both indirect and direct effects: % direct = $(|\Delta S|_{d=1} - |\Delta S|_{d=0})/|\Delta S|_{d=1}$, where $|\Delta S| = |(S_\mu/S_0) - 1|$ is the absolute value of vesicle relative surface deviation from 1. The $d = 1$ subscript signifies that the latter surface deviation occurs when the direct effect is maximally enabled, and thus is a consequence of both direct and indirect effects acting together. Conversely, the $d = 0$ subscript signifies the direct effect is disabled, and only the indirect effect is acting.

Figure 4c						
R	0.5000	1.0000	2.0000	3.0000	4.0000	5.0000
Spherical						
$\|\Delta S\|_{d=1}$	0.1040	0.1831	0.3211	0.4409	0.5482	0.6463
$\|\Delta S\|_{d=0}$	0.0787	0.1401	0.2490	0.3449	0.4314	0.5111
% direct	24.32%	23.49%	22.45%	21.78%	21.30%	20.92%
Deflated 5%						
$\|\Delta S\|_{d=1}$	0.1134	0.2014	0.3563	0.4926	0.6159	0.7300
$\|\Delta S\|_{d=0}$	0.0890	0.1600	0.2872	0.4007	0.5043	0.6008
% direct	21.56%	20.55%	19.40%	18.66%	18.12%	17.69%

Figure 4d					
R	0.1250	0.2500	0.5000	0.7500	1.0000
Spherical					
$\|\Delta S\|_{d=1}$	0.1414	0.1700	0.1963	0.2093	0.2173
$\|\Delta S\|_{d=0}$	0.1125	0.1335	0.1520	0.1608	0.1661
% direct	20.47%	21.46%	22.56%	23.17%	23.57%
Deflated 5%					
$\|\Delta S\|_{d=1}$	0.1625	0.1918	0.2190	0.2325	0.2408
$\|\Delta S\|_{d=0}$	0.1348	0.1569	0.1765	0.1859	0.1916
% direct	17.00%	18.20%	19.40%	20.04%	20.44%

5. Vesicle Breakage

In the paper, to a first approximation, we assume that all of the original vesicles remain intact during competition, with none breaking apart through excessive osmotic stress. When we set an osmotic burst limit of $\Phi = 0.7$ for vesicles, this assumption is valid for Fig. 4 of the paper: pure OA vesicles reached a minimum of $\Phi = 0.77$ in our phospholipid-driven competition simulations, and a minimum of $\Phi = 0.70$ in our osmotic-driven competition simulations. However, Figs 5, 6a and 6c of the paper are affected by this burst criterion.

Supplementary Fig. S2 shows how Figure 5a and 5b of the paper are changed, if we consider that vesicles reaching maximum osmotic tension ($\Phi < 0.7$) fully disintegrate into free lipid monomers, which are in turn absorbed by the remaining vesicles, further boosting their growth.

Whilst in reality, bursting vesicles will not fully convert into free lipid monomer in solution, the lines on the graph can be interpreted as an *upper bound* on the growth of the remaining vesicles, when vesicle bursting is taken into account.

The graph is calculated with the following procedure: (i) The equilibrium lipid concentration in solution $[L]^*$ is calculated for the full initial population, with no vesicles bursting; (ii) The Φ value of all vesicles is evaluated, and the vesicle with lowest the Φ value below $\Phi < 0.7$ is burst. The lipids in the membrane of this vesicle are added to the free lipid monomers in solution; (iii) $[L]^*$ is recalculated for the remaining population, and the procedure is repeated from step (ii) until all surviving vesicles have $\Phi \geq 0.7$, or all vesicles are burst.

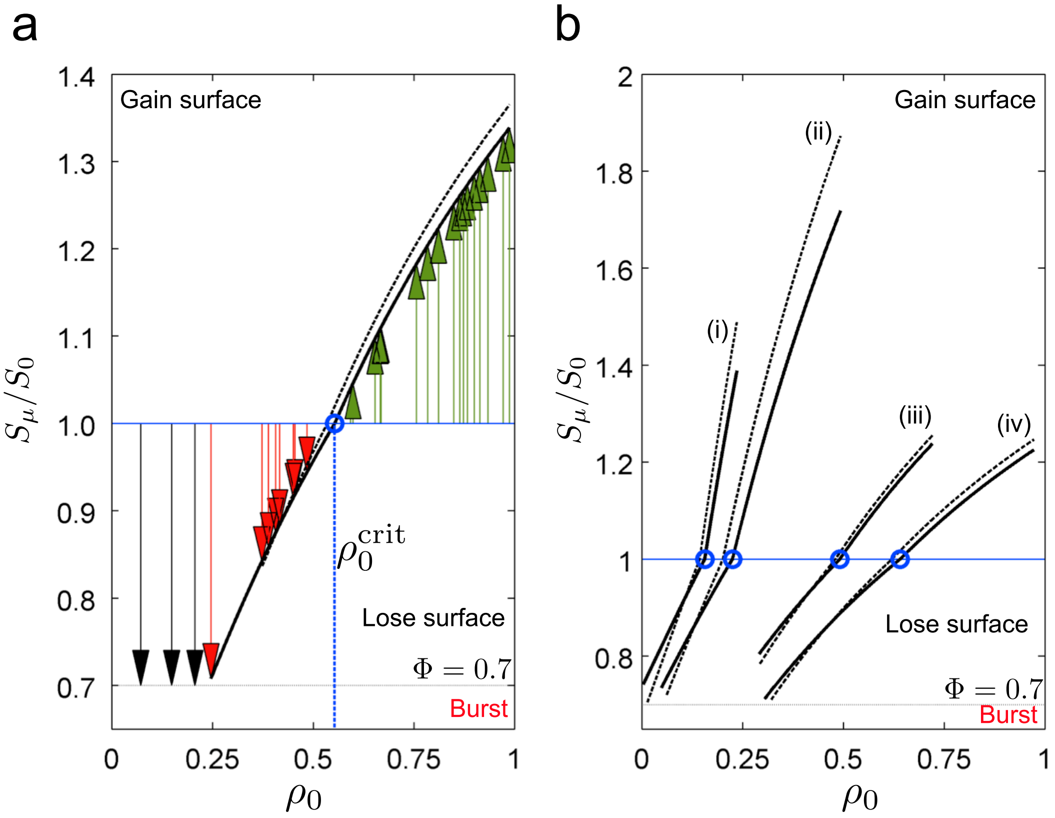

Figure S2: **Lipid competition tipping points, including vesicle breakage.** This figure reports adjustments to Figs. 5a and 5b of the paper, when vesicle breakage is taken into account. **(a)** The three vesicles with the lowest $\Phi < 0.7$ burst (black arrows), which shifts the competition tipping point of the surviving population from $\rho_0^{crit} = 0.584$ to $\rho_0^{crit} = 0.554$, and enhances the maximum relative surface increase of the growing vesicles from around 1.3 to around 1.33. **(b)** Vesicle bursting makes ρ_0^{crit} values of the four vesicle populations slightly lower, and enhances vesicle growth in the populations, especially population (ii).

References

(1) Lonchin, S.; Luisi, P. L.; Walde, P.; Robinson, B. H. *J Phys Chem B* **1999**, *103*, 10910–10916.

(2) Chumpitaz, L. D.; Coutinho, L. F.; Meirelles, A. J. *J Am Oil Chem Soc* **1999**, *76*, 379–382.

(3) Chen, I. A.; Roberts, R. W.; Szostak, J. W. *Science* **2004**, *305*, 1474–1476.

(4) Budin, I.; Szostak, J. W. *Proc Natl Acad Sci U S A* **2011**, *108*, 5249–5254.
